# Action-Oriented Population Nutrition Research: High Demand but Limited Supply

**DOI:** 10.9745/GHSP-D-15-00009

**Published:** 2015-05-27

**Authors:** Judy Pham, David Pelletier

**Affiliations:** ^a^​ Cornell University, Division of Nutritional Sciences, Ithaca, NY, USA

## Abstract

**Background:**

The relatively rapid ascendancy of nutrition and health on policy agendas, along with greater emphasis on accountability and results, has stimulated interest in new forms of research to guide the development and implementation of effective policies, programs, and interventions—what we refer to as action-oriented research. To date, action-oriented research in the nutrition field is thought to be the exception rather than the rule, but empirical evidence to support this claim is lacking.

**Methods:**

We conducted a survey of selected journals in nutrition and public health to assess the extent and nature of population nutrition research published in 2012 that embodied 5 defined characteristics of action-oriented research in relation to: (1) topic(s) of study, (2) processes/influences, (3) actors, (4) methods, and (5) approaches. We identified 762 articles from the 6 selected nutrition journals and 77 nutrition-related articles from the 4 selected public health journals that met our search criteria.

**Results:**

Only 7% of the 762 papers in nutrition journals had at least 1 of the 5 action-oriented research characteristics, compared with 36% of the 77 nutrition-related papers in the public health journals. Of all 80 articles that had at least 1 action-oriented research characteristic, only 5 articles (6.25%) embodied all 5 characteristics. Articles with action-oriented research covered a broad range of topics and processes/influences, including policy, workforce development, and schools, as well as actors, such as program staff, store owners, parents, and school staff. In addition, various research methods were used, such as stakeholder analysis, ethnographic narrative, iterative action research, and decision tree modeling, as well as different approaches, including participant-observer and community-based participatory research.

**Conclusions:**

Action-oriented research represents a small fraction of articles published in nutrition journals, especially compared with public health journals. This reinforces recent calls to expand population nutrition research agendas to more effectively inform and guide the initiation, development, implementation, and governance of policies, programs, and interventions to address the varied forms of nutrition-related problems. With heightened attention to the magnitude and importance of nutrition problems worldwide, there are substantial reasons and opportunities to incentivize and support such expansion.

## Abstract (precis)

Action-oriented research in nutrition, vital to guiding effective policies and programs at scale, is greatly underrepresented in public health journals and, even more so, in nutrition journals.

## INTRODUCTION

Nutrition is now recognized as a major cause or contributing factor to a wide range of diseases and to the global burden of disease in developed as well as developing countries.^1^ In addition to its role in morbidity and mortality, poor nutrition can increase health care costs, and it contributes negatively to cognitive and motor development, school performance, economic productivity, and national economic growth.^2^^–^^6^ For these reasons, and as a fundamental aspect of human rights and equity, nutrition has risen on the agendas of international organizations, governments in developed and developing countries, the private sector, and in popular culture.^7^^–^^9^ This rapid ascendancy of nutrition on policy agendas has some similarities to the ascendancy of global health that began a decade or two earlier.^10^ One of the key features they share in common is a greater concern for accountability and results.^11^^–^^13^

The concern for accountability and results has revealed a yawning gap in funding between mechanistic or efficacy research and research aimed at delivering results at scale. For instance, the *Lancet* child survival series estimated in 2003 that global child mortality could be reduced by two-thirds through universal coverage of existing interventions,^14^ but a separate analysis revealed that 97% of child health research grants are focused on developing new interventions rather than enhancing the delivery of existing interventions.^15^

Universal coverage of existing interventions could reduce child mortality by two-thirds, but 97% of child health research grants focus on developing new interventions.

The recognition of these gaps and the desire to produce results at scale have generated interest in newer forms of research to guide the initiation, development, implementation, and governance of effective policies and programs. Research for these purposes involves research questions, designs, methods, partnerships, and funding that are distinct from the well-developed forms of research, such as randomized controlled trials, used in discovery or efficacy research.^16^ The newly emergent forms of research come under a variety of labels such as implementation or delivery science,^17^ translational research,^18^^,^^19^ community-based participatory research,^20^ action research,^21^ developmental evaluation,^22^ and engaged or prospective policy research,^23^ among others.^24^ Although all these types of research share a desire to create knowledge that can inform and guide solutions to health and nutrition problems, they differ markedly in the geographic scale (communities and countries to global institutions), objects of inquiry (e.g., health workers, managers, mHealth, training and supervision approaches), disciplinary theories and methods (anthropology and management to economics and political science), and the journals in which the findings are published. The advent of *Global Health: Science and Practice* is one manifestation of this growing interest in producing and disseminating practice-oriented knowledge and experience.

New forms of research, referred to here as action-oriented research, aim to guide effective health and nutrition policies and programs.

While this diversity in action-oriented health and nutrition research is a potential strength and is appropriate given the diversity in contexts where action must take place, it also poses a danger in that results from these emergent forms of research may remain highly particularistic and contextual.^25^ If these new forms of research are to gain legitimacy and form a coherent and cumulative body of knowledge about how to address health and nutrition problems in diverse contexts, there will need to be some parallel intellectual work to develop, refine, and share integrative knowledge, theory, frameworks, and methods.^16^

To this end, we recently published a framework for organizing and ultimately advancing the knowledge, principles, and practices related to action-oriented research in population nutrition, most of which deal with the implementation of policies, programs, and interventions.^26^ Although that paper focused on nutrition, the framework and principles are equally relevant for global health more broadly and for other domains. The present paper provides a brief overview of the framework and reports on the results of a literature search designed to assess the extent to which and how researchers are currently working at these research frontiers in the case of nutrition.

## A FRAMEWORK FOR DEFINING ACTION-ORIENTED NUTRITION RESEARCH

Implementation research and the other newly emergent forms of research in health and nutrition noted above are actually part of a larger transformation in science that is underway at the societal level. In the sociology of science literature, this transformation has been famously termed “Mode 2” knowledge production.^27^^,^^28^ According to this literature, the conventional production of scientific knowledge (“Mode 1”) takes place primarily in academic and scientific institutions and is governed by the norms of scientific disciplines, whereas Mode 2 knowledge production takes place through greater interaction with communities, government actors, NGOs, and/or private-sectors actors. Mode 2 knowledge production is considered an emergent and socially robust form that complements Mode 1 and is especially needed for addressing complex social problems. Its emergence is due to external (societal) trends and pressures, such as the demand for greater accountability, as well as internal forces and incentives within universities and other research institutions.

Action-oriented research involves greater interaction with communities, government, NGOs, and the private sector than conventional science.

According to these authors, Mode 2 knowledge production differs from that of Mode 1 in several ways:

- It takes place in the context of application or problem solving (versus theoretical or strictly academic contexts).
- It is transdisciplinary (versus disciplinary or even interdisciplinary), drawing upon whichever disciplinary and contextual knowledge is needed to address the problem at hand.
- It is heterogeneous in its sites, including mission-focused research centers, government agencies, think tanks, nonprofit agencies, communities of practice, epistemic communities, and community organizations (versus universities and research centers).
- It arises from mutual interaction among these actors and sites (versus interaction mainly among academic peers).
- It involves novel forms of quality control based on economic, political, social, ethical, and utility criteria (versus discipline-based norms and peer review alone).
- As a result of the social interaction, it is reflexive (embracing of multiple perspectives on problem solving versus search for a single truth) and more intentionally socially accountable (versus accountable only to scientific and disciplinary norms).

Based on this earlier work, we proposed 6 dimensions or tendencies that might define action-oriented population nutrition research (Table 1).^26^ These dimensions resonate well with the current understanding of implementation research as elaborated elsewhere,^24^^,^^29^ but the present study did not limit itself to that focus.

**TABLE 1.** Six Dimensions of Action-Oriented Population Nutrition Research^a^

**Dimension**	**Conventional Research**	**Action-Oriented Research**
**Why we study**	To create generalizable or fundamental knowledge that answers scientific questions	To create knowledge that can help identify, characterize, and solve practical problems of concern to stakeholders, organizations, communities, or publics at various scales
**What we study (topics)**	Nutrients, food and nutrient intake, consumer behavior, determinants and consequences of nutritional variation, efficacy of interventions	Food and nutrition issues, causes, and solutions in a broader social and action context, including food systems, social and public health programs and policies; processes of policy agenda setting, governance, development, implementation, scaling-up, and evaluation; and community and organizational behavior and change processes
**Who we study (actors)**	Mothers, infants, children, individuals, consumers, patients	Policy makers, analysts, managers, implementers, frontline workers in the public sector; global, national, state, and local leaders and members of communities, civil society organizations, universities, networks, and coalitions; global, national, state, and local private-sector actors and entities, citizens, academics
**How we study: methods**	Measurements of knowledge, attitudes, beliefs, behavior, biology, individual and environmental characteristics, and their interrelationships, using a limited range of quantitative and qualitative methods	More eclectic range of qualitative and quantitative methods to inquire into the new topics noted above, including mixed methods, social network analysis, discourse analysis, narrative policy analysis, Q methodology, process tracing, stakeholder analysis and influence mapping, program impact pathways, organizational ethnography, systems dynamics group modeling
**How we study: approaches**	Generally detached, objectivist, positivist, reductionist, behaviorist, hypothesis testing	More engaged, participatory, action research, community-based participatory research, participant-observer, reflection in action, embedded, critical, social construction, emergent, systems- and complexity-oriented
**Disciplinary foundations**	Nutritional sciences, epidemiology and biostatistics, biomedicine, psychology, social psychology, consumer behavior	Transdisciplinary, drawing upon our traditional disciplines but also with a greater role for economics, sociology, anthropology, policy analysis, law, urban planning, political science, organizational behavior, management sciences, and systems sciences

a In many cases, the distinctions shown in this table are a matter of degree or emphasis rather than discrete categories. Individual studies or research programs may possess many or few of these characteristics, to a greater or lesser extent.

Reprinted and adapted with permission from Pelletier et al., 2013 in *Advances in Nutrition*.^26^ Copyright 2013 by American Society for Nutrition.

The 6 dimensions are:

1. **Why we study (“why”):** the central feature of action-oriented research, influencing all other dimensions. The primary motivation of action-oriented research is to help identify, characterize, and solve practical problems.
2. **What we study (“topics”):** examine food and nutrition issues in a broader context beyond individual-level biology and behavior, including a focus on food systems, social and public health programs and policies, organizational behavior, and change processes at various levels of social organization
3. **Who we study (“actors”):** also moves beyond the individuals directly affected (mothers, infants, consumers, etc.) and instead studies those engaged in food and nutrition efforts directly or indirectly, such as government agencies, policy makers, frontline workers, civil society organizations, academic institutions, and private-sector actors
4. **How we study in terms of methods (“methods”):** a range of qualitative and quantitative methods that may include but go beyond the conventional methods of focus groups, interviews, and/or surveys that measure knowledge, attitudes, beliefs, behavior, and biology
5. **How we study in terms of approaches (“approaches”):** involves interaction between researchers and various social actors, and thus can be described as engaged and participatory as opposed to detached and seeking objectivity
6. **Disciplinary foundations (“disciplines”):** draws upon the conventional disciplines (e.g., nutritional sciences, epidemiology, and behavioral psychology) but also includes perspectives, theories, and collaboration from other disciplines, such as anthropology, economics, law, policy analysis, and management. Importantly, the motivation of creating actionable knowledge often leads to a transdisciplinary orientation, in which the disciplines, theories, and constructs used in a particular case are defined based on the characteristics of the problem in a given context, rather than the disciplinary norms of the researchers and/or their institutions.

According to Pelletier et al.,^26^ action-oriented research currently is the exception rather than the rule in population nutrition research, and there is a need to expand in these directions in order to develop more effective, appropriate, and sustainable responses to food and nutrition problems. The purpose of this paper is to provide systematic empirical support for that claim.

## METHODS

Based on the 6-dimension framework, we developed and applied a literature coding system to peer-reviewed literature published in 2012 from selected journals in the areas of nutrition and public health. The public health journals were included to provide a contrast with the nutrition journals. We modeled our coding system after a methodology applied to examine the use of social-ecological approaches in the design of health promotion interventions over a 20-year period.^30^ We also consulted the Preferred Reporting Items for Systematic Reviews and Meta-Analyses (PRISMA) statement for guidance.^31^

**Figure:**
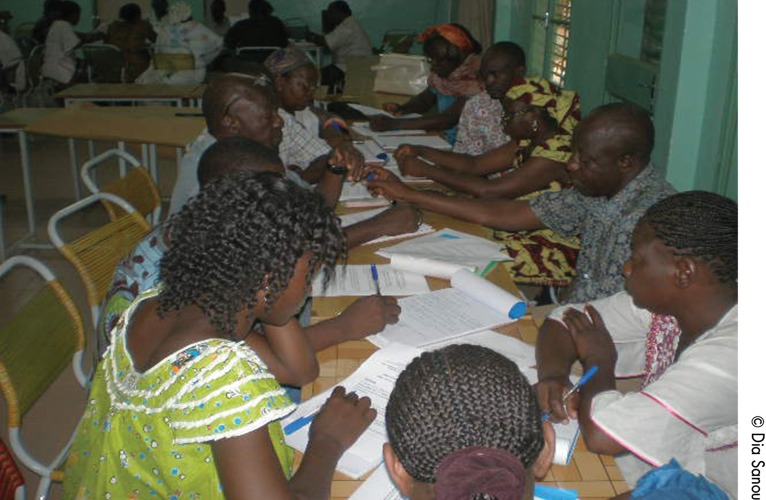
Participants in Mali analyze data on infant and young child feeding practices during a community diagnosis. Such participatory and action-oriented research methods tend to examine health problems in a broader context and involve community actors in the research process itself.

Purposeful sampling was used to focus on the journals most likely to publish action-oriented research, through consultation with nutrition colleagues familiar with the field.

The nutrition journals selected were:

- Ecology of Food and Nutrition
- Journal of Nutrition Education and Behavior
- Maternal and Child Nutrition
- Journal of Nutrition
- Public Health Nutrition
- International Journal of Behavioral Nutrition and Physical Activity

The public health journals selected were:

- American Journal of Public Health
- Health Policy and Planning
- Social Science & Medicine
- Journal of School Health

We used the Thomas Reuters Web of Science database to screen all articles published in 2012 in these journals. After eliminating papers not focused at the population level and/or not focusing on nutrition (described below), the remaining articles were sorted by using a coding sheet initially based on the 6 action-oriented research dimensions. The coding was refined using successive trial runs, and the final coding sheet included 5 dimensions that could be systematically and objectively identified in the articles.

The final 5 characteristics, which required some modifications to the original 6 dimensions to embrace the actual diversity found in the articles, comprised: (1) topic(s) of study, (2) processes/influences, (3) actors, (4) methods, and (5) approach. Characteristics 3, 4, and 5 are from the original framework. We eliminated the “why” and “disciplines” dimensions from the original framework due to limitations in our ability to identify them without making assumptions about authors’ intentions or the nature or extent of any transdisciplinary orientation. Characteristics 1 and 2 in our study corresponded to the “topics” dimension from the original framework but was divided into 2 categories to distinguish papers that focused on entities (e.g., interventions and public programs) from papers that focused on processes or influences (e.g., policy development, community or organizational change).

Nutrition articles were coded based on 5 action-oriented research characteristics: topic, processes/influences, actors, methods, and approach.

For the nutrition journals, we first eliminated papers that focused narrowly on topics such as measurement of body mass index, birth weight (without any nutritional correlates), pregnancy cravings, tobacco, physical activity, disease, aging, oral health, or housing, based on article titles and abstracts when necessary. In the second stage, the remaining papers were hand-sorted with the coding sheet by title, abstract, and full text, as necessary. Those embodying none of the action-oriented characteristics were also eliminated. One reviewer (JP) tabulated the titles and abstracts for the final papers included in our analysis according to each characteristic and noted the reason(s) for the tabulation, while another reviewer (DP) reviewed all tabulations. In cases where there was disagreement, the reviewers met to discuss until they reached agreement.

For the public health journals, we identified nutrition-related papers by using the following search terms in the topic search field, using the Web of Science database: *nutrition OR malnutrition OR undernutrition OR food OR obesity OR micronutrient OR supplementation OR nutrient OR diet OR hunger*. Those papers not meeting any of these search criteria were considered non-nutrition articles and were not considered further. The articles meeting the nutrition search criteria were subjected to the same coding and review protocol as the nutrition journal papers, to identify the subset with at least 1 action-oriented research characteristic.

## RESULTS

After employing our first-stage elimination strategies in which we excluded articles in nutrition journals with a narrow topic and articles in public health journals that did not meet our nutrition search terms criteria, we identified and reviewed a total of 839 articles (762 from nutrition journals, 77 from public health journals). Overall, less than 10% of these articles possessed at least 1 action-oriented research characteristic and were ultimately included in our analysis (n = 52 from nutrition journals, n = 28 from public health journals) (Table 2). (See the supplementary material for a bibliography of the papers with at least 1 action-oriented research characteristic, which were included in our analysis.)

Less than 10% of nutrition articles published in 2012 embodied at least 1 action-oriented research characteristic.

**TABLE 2.** Nutrition-Focused Papers With Action-Oriented Research Characteristics Published in 2012 in Nutrition and Public Health Journals

**Journal Name**	**Total No. of Nutrition Papers in 2012**	**No. (%) of Papers With **≥**1 Action-Oriented Characteristic^a^**	**No. (%) of Papers per Action-Oriented Characteristic^b^**				
			**Topic(s) of study**	**Processes/ Influences**	**Actors**	**Methods**	**Approach**
**Nutrition Journals**						
Ecology of Food and Nutrition	19	4 (21.1)	3 (75.0)	4 (100.0)	1 (25.0)	2 (50.0)	2 (50.0)
Journal of Nutrition Education and Behavior	90	13 (14.4)	6 (46.2)	11 (84.6)	6 (46.2)	3 (23.1)	4 (30.8)
Maternal and Child Nutrition	47	5 (10.6)	5 (100.0)	4 (80.0)	5 (100.0)	2 (40.0)	3 (60.0)
Journal of Nutrition	308	7 (2.3)	6 (85.7)	5 (71.4)	1 (14.3)	0 (0.0)	0 (0.0)
Public Health Nutrition	252	22 (8.7)	15 (68.2)	17 (77.3)	9 (40.9)	9 (40.9)	7 (31.8)
International Journal of Behavioral Nutrition and Physical Activity	46	1 (2.2)	1 (100.0)	1 (100.0)	1 (100.0)	0 (0.0)	0 (0.0)
**Subtotal**	**762**	**52 (6.8)**	**36 (69.2)**	**42 (80.8)**	**23 (44.2)**	**16 (30.8)**	**16 (30.8)**
**Public Health Journals**						
American Journal of Public Health	24	8 (33.3)	8 (100.0)	7 (87.5)	3 (37.5)	4 (50.0)	0 (0.0)
Health Policy and Planning	4	4 (100.0)	4 (100.0)	4 (100.0)	2 (50.0)	4 (100.0)	3 (75.0)
Social Science & Medicine	26	7 (26.9)	7 (100.0)	5 (71.4)	3 (42.9)	4 (57.1)	3 (42.9)
Journal of School Health	23	9 (39.1)	9 (100.0)	9 (100.0)	4 (44.4)	4 (44.4)	2 (22.2)
**Subtotal**	**77**	**28 (36.4)**	**28 (100.0)**	**25 (89.3)**	**12 (42.9)**	**16 (57.1)**	**8 (28.9)**
**TOTAL**	**839**	**80 (9.5)**	**64 (80.0)**	**67 (83.8)**	**35 (43.8)**	**32 (40.0)**	**24 (30.0)**

a Percentages are of total nutrition papers per journal.

b Percentages are of total nutrition papers per journal with ≥1 action-oriented research characteristic.

Of the 80 articles that had at least 1 action-oriented research characteristic, 5 articles (6.25%) embodied all 5 characteristics. There was a notable difference in the frequency of action-oriented research articles between the nutrition journals (7% with at least 1 characteristic) and the public health journals (36%).

Public health journals had a higher proportion of action-oriented nutrition research articles than nutrition journals.

### Action-Oriented Research in Nutrition Journals

Of the 52 articles from nutrition journals that had 1 or more action-oriented research characteristic, 69% fulfilled the criteria for “topic(s) of study,” 81% for “processes/influences,” 44% for “actors,” 31% for “method,” and 31% for “approach” (Table 2). Only 2% of the articles embodied all 5 characteristics while 17% embodied only 1 characteristic, 35% embodied 2 characteristics, 25% embodied 3 characteristics, and 21% embodied 4 characteristics (Figure).

**FIGURE.**
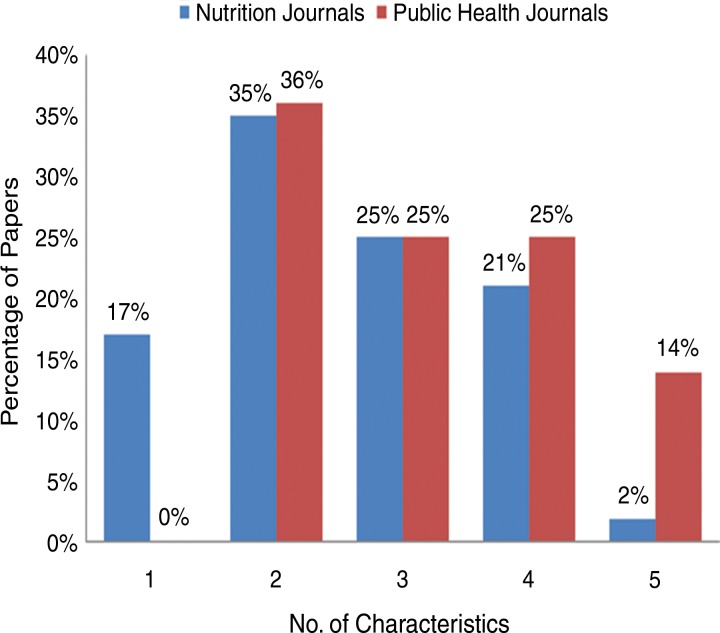
Number of Action-Oriented Research Characteristics in Nutrition Papers by Journal Type (N=80)

The “topic(s) of study” and “processes/influences” covered a broad range of topics, including national policy, workforce development, programs, and schools, among others (Table 3). One illustrative example in workforce development was an article titled, “Using video narratives of women’s lived experience of breastfeeding in midwifery education: exploring its impact on midwives’ attitudes to breastfeeding,” which studied midwifery breastfeeding counseling as the topic of study and the midwives’ attitudes toward breastfeeding as the outcome.^32^ Another article, “Public health nutrition workforce development in seven European countries: constraining and enabling factors,” studied public health nutrition workforce development in 7 European countries as the topic of study and constraining and enabling factors in terms of the policy environment, public health organizations, and workforce compensation as the processes/influences.^33^

**TABLE 3.** Illustrations of Action-Oriented Research Characteristics of “Topic(s) of Study” and “Processes/Influences” in Nutrition Journals

**Key Topics**	**Topic(s) of Study**	**Processes/Influences**
National policy	US, Ireland, UK, dietary guidelines, growth charts, nutrition in child-care settings, revision process, development process, communications initiatives	Translation at local/regional levels, barriers to and extent of adoption, revisions, evaluation, practitioners’ understanding of growth charts, cost implications, public health expenditures
Workforce development	Certification programs, register of nutritionists, required core functions, teaching and training initiatives, midwifery breastfeeding counseling	Constraining/enabling factors, stakeholder consensus on core functions, incorporating cognitive-behavioral techniques into breastfeeding counseling
Programs	Public-private partnerships, church-based, transdisciplinary platforms for interventions, e.g., health, agriculture, market, social protection	Partnership opportunities, changed program practices, cost-effectiveness, challenges for dissemination, new evaluation framework, development of young adult obesity program based on community-based participatory research, implementation fidelity
Schools	Nutrition guidelines, school gardens	Instructional process, decision making, environment, food service offerings, food preparation practices
Global	Immigrant experience, political instability, economic instability, drought, global food system, regional early warning systems	Food nostalgia and cultural symbolism, household provision of care for people living with HIV/AIDS, real cost of food, policy options to improve food security, ability to predict food crises
Other	Media content, employers’ attitudes toward mother-friendly work environments, breastfeeding peer support services, grocery store marketing and promotion, WIC-authorized stores	Confusion resulting from media news reporting, eating maps, food store stocking and pricing behavior changes after food assistance program changes, employer readiness to provide breastfeeding accommodations, marketing on packaging

Abbreviation: WIC, Special Supplemental Nutrition Program for Women, Infants, and Children.

The “actor” characteristic in the action-oriented research articles included program staff, private-sector stakeholders, store owners, government employees, parents, school staff, and peer supporters, among others (Table 4). Various research “methods” were used, including stakeholder analysis, onsite receipt collection, ethnographic narrative, and iterative action research (Table 4), and “approaches” consisted of participant-observer, engaged, complexity-aware and prospective policy research, and community-based participatory research (not shown).

**TABLE 4.** Illustrations of Action-Oriented Characteristics of “Actors” and “Methods” in Nutrition Journals

**Actors**	**Methods**
Child care professionals	Consultative workshops
Clinical staff	Emerging policy options with stakeholder input
Community health workers	Health economic analysis
Community leaders	Impact pathways
Food assistance program staff	Implementation pathways
Food service employees	Immersion-observation
Government authorities and advisors	Iterative action research via workshops
Health professionals	Onsite receipt collection
Peer supporters	Policy review
Private-sector employers	Simulation of food intake patterns
Program implementers	Stakeholder analysis
School staff, parents, volunteers	Systematic Internet review
Stakeholders, i.e. academics, practitioners	Thematic analysis
Store owners/managers	

### Action-Oriented Research in Public Health Journals

Of the 28 articles in public health journals with 1 or more action-oriented research characteristic, 100% fulfilled the criterion for “topic(s) of study,” 89% for “processes/influences,” 43% for “actor,” 57% for “method,” and 29% for “approach” (Table 2). None of these papers embodied only 1 action-oriented research characteristic, 36% embodied 2 characteristics, 25% embodied 3 characteristics, 25% embodied 4 characteristics, and 14% embodied all 5 characteristics (Figure).

In the public health journals, the “topic(s) of study” and “processes/influences” covered a broad range of topics, as in the nutrition journals, including policy and legislation, schools, and programs (Table 5). The “actor” category included midwives, school principals, community members, and NGOs, among others (Table 6). Some examples of the “methods” included change-making process analysis, exploratory case study, consultative workshops, and decision tree modeling, and the “approaches” comprised participant-observer, community-based participatory research, engaged, and prospective policy research (not shown).

**TABLE 5.** Illustration of Action-Oriented Research Characteristics of “Topic(s)” and “Processes/Influences” in Public Health Journals

**Key Topics**	**Topic(s) of Study**	**Processes/Influences**
Policy and legislation	Changes in WIC policy, state childhood obesity policies, national nutrition agenda setting, policy formulation and implementation	Predictors of enactment, instruments prescribed to influence school food environment, strategies used to move nutrition agenda forward, enabling/inhibiting factors, levels of commitment, policy diffusion from state- to district-level
Schools	Elementary/high schools, school beverage shipments, school bus advertising, school-based obesity policy, wellness policy requirements, state department of education policy and structural changes to improve nutrition	Beverage industry self-regulation, sugar-sweetened beverage availability, acceptability of specific intervention strategies, changes in wellness policies before and after federal mandates, changes in food options, food service finances, implementation and awareness of guidelines
Programs	Outcomes and cost of community-based management of acute malnutrition, procedural programs to create healthy environments for vulnerable populations, promotional tool for healthy body image	Implementation processes, lessons learned, cost-effectiveness, extent of cooperation, population reach, perceived potential of tool
Other	Food advertising, language of midwives, GDP/Gini Index/GII, climate change, international human rights obligations regarding rights to food and health	National approaches to addressing food insecurity, impacts on gender inequality, global distribution of obesity, impacts on household decision making

Abbreviations: GDP, Gross Domestic Product; GII, Global Innovation Index; WIC, Special Supplemental Nutrition Program for Women, Infants, and Children.

**TABLE 6.** Illustrations of Action-Oriented Characteristics of “Actors” and “Methods” in Public Health Journals

**Actors**	**Methods**
Community members	Change-making process analysis
Frontline staff	Coding of media photos
Government officials	Consultative workshops
Midwives	Decision tree modeling
NGOs, donors, civil society	Discourse analysis
Physical education teachers	Document analysis
Private sector	Exploratory case study
Program administrative staff	Information gathering from practitioners
School health advisory councils	Observation
School principals	Project performance framework
	Systematic review
	Theoretical policy science typology
	Wellness policy coding scheme

## DISCUSSION

*This paper argues that much health policy [research] wrongly focuses attention on the content of reform, and neglects the actors involved in policy reform (at the international, national and sub-national levels), the processes contingent on developing and implementing change and the context within which policy is developed. Focus on policy content diverts attention from understanding the processes which explain why desired policy outcomes fail to emerge*.^34^

The above quote from 20 years ago refers to the state of research on health policy in developing countries, but it could just as well apply to much of population nutrition research today. The present study, consistent with the claims made by Pelletier et al. in the earlier paper that outlined a framework for defining action-oriented nutrition research,^26^ finds a paucity of research on the actors, processes, and contexts within which nutrition policy (or actions in general) is developed and implemented, suggesting that the majority of nutrition research currently being published by nutrition and public health academics contains a relatively narrow range of topics, methods, and approaches. Specifically, fewer than 10% of the reviewed nutrition articles embodied at least 1 action-oriented research characteristic, which itself is a minimalist criterion. Those that did meet this criterion, however, exhibited a rich array of action-oriented research topics, processes/influences, methods, and approaches to study, indicating that this type of research is feasible and can be expanded in the future.

The small set of nutrition research articles that were action-oriented exhibited a rich variety of topics, methods, and approaches, indicating such research is feasible.

The sizeable difference between the proportion of action-oriented research papers published in nutrition journals (only 7% of reviewed papers had at least 1 action-oriented research characteristic) versus public health journals (36%) suggests that articles in public health journals are more likely to be engaged in problem-solving research and to have expanded their research questions, approaches, and methods, compared with those published in nutrition science journals. It is unclear from the present study whether this difference reflects greater receptivity to action-oriented research papers in public health journals or a preference for action-oriented researchers to publish in those journals. Given the importance of action-oriented research for informing and guiding solutions to high-burden and highly salient food and nutrition problems, the present study suggests there is an undersupply of such research, especially in nutrition journals.

In fact, for at least the last 2 decades, the need for more action-oriented research has been recognized^35^^–^^37^ to answer such problems as how to deliver solutions effectively and sustainably at large scale,^38^ how to increase demand for and use of existing nutrition services and products,^39^ and how to ensure relevance of nutrition research to policy makers and program implementers.^40^ Most recently, the New York Academy of Sciences, in collaboration with the World Health Organization, launched a solution-oriented global research agenda for nutrition,^37^ and a new society for implementation research on nutrition is being formed for that purpose.^41^

**Figure:**
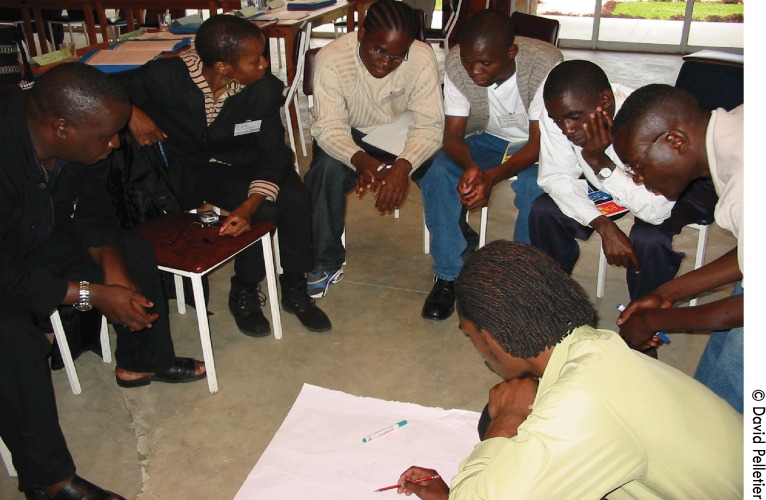
In Ghana, a group of young men map factors and locations in their community that place youth at risk for HIV. This type of youth action research trains young people in the research process, including using their findings to inform direct change in their communities.

The literature offers several explanations for this undersupply of action-oriented nutrition research. Allen and Gillespie discuss nutrition researchers’ scientific predisposition to address questions of efficacy rather than effectiveness^42^; Garrett points toward nutrition researchers’ limited familiarity with other disciplines and the contributions they can make^40^; Shekar cites the real and/or perceived lack of funding for such research^38^; Berg examines the academic culture and training that inhibit interest in applied research^43^; and nutrition stakeholders within sub-Saharan Africa identify a need for better governance of nutrition research, alignment of nutrition research funding with priorities identified within the region, and capacity development for nutrition research. The literature and experience of health policy research concurs with these explanations and also identifies a heavy reliance on international funding for research, an excessive focus on the direct utility of findings from specific studies, and a tendency to undervalue contributions from social sciences.^44^

The interpretation of the findings in this paper requires 2 important qualifications. First, as detailed in the original paper outlining the action-oriented research framework,^26^ the focus on action-oriented research is not to dismiss or discount the importance of conventional nutrition research. Rather, as noted elsewhere,^24^ the design, implementation, and sustainability of effective solutions to nutrition problems require the integration of knowledge from both forms of research, so that knowledge of intervention efficacy can be combined with knowledge of and strategies for agenda setting, commitment building, policy and program formulation and implementation, and related activities.

Effective solutions to nutrition problems require application of knowledge from both action-oriented and conventional nutrition research.

Second, the sparseness of action-oriented research in nutrition journals raises the question: Does it matter? Perhaps what really matters is that nutrition researchers and/or researchers from other disciplines are doing action-oriented research and publishing it in whichever journals are receptive to such research. This may be a practical strategy in the short-term, if the current perspectives and priorities in nutrition science journals are not receptive. However, this short-term strategy would raise concerns if it inhibits the continued intellectual growth and policy/programmatic relevance of the nutrition research community itself or reflects an institutionalized resistance to such growth. In the long-run, it will be important for action-oriented research to become more mainstreamed within nutrition curricula, research agendas, and donor funding. The focus and content of nutrition journals play important gatekeeper, incentivizing, and symbolic functions in that mainstreaming process.

### Limitations

The present study has several technical limitations. First, it focused only on a sample of nutrition and public health journals and only on papers published in 2012, in order to provide an initial sense of the current research tendencies. A more comprehensive bibliometric survey would be useful to ensure broader coverage and provide a baseline for examining trends over time. Second, the low frequency of action-oriented nutrition research articles may reflect editorial preferences of the journals rather than the actual volume of such research. The possibility of publication bias cannot be examined with these data sources alone. Third, the data for this research are based on analysis of material provided in the papers themselves, rather than on direct communication with authors, which could have resulted in some inaccurate coding. While acknowledging these issues, it also seems likely that the overall findings are rather robust to such limitations. Finally, while most of the action-oriented research papers identified through the bibliometric search in this paper are focused on topics, processes, and/or actors related to implementation of policies, programs, or interventions, it is important to note that search terms such as “implementation” or “delivery” were not employed in this study. Given the significant and growing interest in implementation research per se, a high priority for future research is to conduct a more comprehensive survey of the literature to establish benchmarks and directions for this growing field of inquiry.

## CONCLUSION

Action-oriented research represents a relatively small fraction of papers published in nutrition journals, even when the search is restricted to the journals most likely to publish such research and when a minimal set of criteria is applied. Public health journals, in contrast, are far more likely to publish nutrition research with action-oriented characteristics. Existing action-oriented research exhibits a rich array of topics, methods, and approaches, indicating that this type of research is feasible and can be expanded in the future. With heightened attention to the magnitude and importance of nutrition problems worldwide and the emphasis placed on accountability and results, there are substantial opportunities and obligations for all of parties in the research enterprise, from research institutions and graduate training programs to journals and research funders, to incentivize and support such an expansion.^45^

## Notes

**Peer Reviewed**
